# Prostate cancer: a risk factor for COVID-19 in males? A protocol for systematic review and meta analysis

**DOI:** 10.1097/MD.0000000000022591

**Published:** 2020-10-23

**Authors:** Ruiyu Mou, Xinyao Jin, Wenjie Li, Mingxin Wu, Xiaodi Liu, Zhao Liu, Shanqi Guo, Xiaojiang Li, Yingjie Jia

**Affiliations:** a First Teaching Hospital of Tianjin University of Traditional Chinese Medicine; b Tianjin Key Laboratory of Translational Research of TCM Prescription and Syndrome; c Evidence-based Medicine Center of Tianjin University of Traditional Chinese Medicine, Tianjin, China.

**Keywords:** COVID-19, meta-analysis, prostate cancer, protocol, risk factor, systematic review

## Abstract

**Introduction:**

COVID-19 is now a global pandemic. Although there are very few studies describing the characteristics of SARS-CoV-2 infections in patients with prostate cancer, these patients are likely to be more susceptible to COVID-19 than healthy people because of their immunosuppressed state. However, there is no evidence that prostate cancer is a risk factor for COVID-19.

**Methods:**

We searched the Wanfang database, the China Science Journal Citation Report (VIP database), the China National Knowledge Infrastructure (CNKI), Web of Science, EMBASE, PubMed, and the Cochrane Library for studies related to the topic. We designed a standardized data extraction sheet and used Epidata software 3.1 for data extraction. In accordance with the Cochrane 5.1.0 standard, both a quality assessment and a risk assessment were carried out for the research meeting the inclusion criteria. The data were analyzed using Revman 5.3 and Stata 13.0 software.

**Results:**

The study integrated existing research findings and a meta-analysis of the data to investigate the prevalence of prostate cancer in males infected with SARS-CoV-2 and the adverse clinical outcomes in male patients with or without COVID-19.

**Conclusion:**

The results of this research may provide a basis for judging if prostate cancer is a risk factor for males infected with SARS-CoV-2, and the findings can effectively help to prevent COVID-19 in patients with prostate cancer.

**Ethics and dissemination:**

Ethics approval is not required for this systematic review as it will involve the collection and analysis of secondary data. The results of the review will be reported in international peer-reviewed journals

**PRORPERO registration number:**

CRD42020194071.

## 1 Introduction

In 2019, the World Health Organization declared coronavirus disease (COVID-19) to be a global epidemic. Since then, the global outbreak of COVID-19 has seriously threatened human health.^[1–3]^ According to data from the Johns Hopkins Coronavirus Resource Center, as of July 27, 2020, SARS-CoV-2 has caused more than 16 million infections and more than 640,000 deaths.^[4]^ Prostate cancer has a higher incidence of male malignant tumors and seriously harms men's health. According to the 2018 Global Cancer Report, there were 9.5 million new cases of prostate cancer and 5.4 million new deaths worldwide, and its global incidence is second only to lung cancer.^[5]^

A previous study has shown that there was no difference in SARS-CoV-2 infection rates between the sexes;^[6]^ whereas, another study found that 58% of 1099 patients with COVID-19 were male.^[7]^ Among critically ill patients, the prevalence of COVID-19 was found to be higher in males than females.^[8]^ Moreover, men with COVID-19 have worse clinical prognoses than women with the disease.^[9]^ A total of 9,280 COVID-19 records were collected from hospitals in Veneto, Italy, and they showed that, overall, men had more severe complications, were more likely to be hospitalized, and had poorer clinical outcomes than women. Furthermore, the risk of SARS-CoV-2 infection in cancer patients is higher than in healthy individuals.^[10]^ Therefore, we hypothesized that infection with SARS-CoV-2 may have a gender bias, and men may be more likely to be infected.

Since the outbreak of COVID-19, a large number of studies have reported the clinical, radiological, and virologic characteristics of the disease, as well as the risk factors for serious disease and death, including the age of diagnosis, complications of SARS-CoV-2 infection, ICU admission rate, adverse reactions caused by CT, high SOFA score, and mechanical ventilation requirements. Although there are insufficient studies to accurately describe the characteristics of SARS-CoV-2 infections in patients with prostate cancer, it is possible that these patients are more likely to be infected with SARS-CoV-2 because of their immunosuppressed status. Therefore, the aim of our study was to determine the prevalence of prostate cancer in males infected with SARS-CoV-2 and the adverse clinical outcomes in male patients with and without COVID-19.

## 2 Methods

### 2.1 Protocol and registration

We completed the systematic review protocol agreement in the international prospective register of systematic reviews (PROSPERO, https://www.crd.york.ac.uk/PROSPERO); its unique registration number is CRD42020194071. The review report of the experiment is based on the recommendation from the Cochrane Handbook for Systematic Review of interventions and the Reporting Items for Systematic Reviews and Meta-Analyses Protocols (PRISMA-P). Any modification to the scheme will be recorded in the comprehensive review and the revised scheme will be subsequently updated.

### 2.2 Eligibility criteria

#### 2.2.1 Types of study

The studies included cohort studies, case-control studies, cross-sectional studies, randomized controlled trials, and nonrandomized controlled trials. The following studies will be excluded: publications using the same or overlapping data, reviews or meta-analyses, letters to editors, comments, editorials, case reports, studies which do not include useful data, and studies with sample sizes of less than 10.

#### 2.2.2 Type of participant

Participants must meet the following conditions: male patients confirmed with COVID-19 based on positive COVID-19 RNA reverse-transcription polymerase chain reaction (RT-PCR) results; male patients confirmed with COVID-19 based on clinical diagnosis, according to clinical guidelines; male prostate cancer patients confirmed with COVID-19 based on positive COVID-19 RNA RT-PCR results; male prostate cancer patients confirmed with COVID-19 based on clinical diagnosis, according to clinical guidelines.

#### 2.2.3 Type of intervention

The only type of intervention in this study was to exclude male patients without COVID-19.

#### 2.2.4 Types of outcomes

##### 2.2.4.1 Primary outcomes

The primary outcomes of the study include the following two aspects. The first is to clarify the prevalence of prostate cancer among men diagnosed with COVID-19. The second aspect is to study the differences in the clinical outcomes of COVID-19 male patients with or without prostate cancer.

##### 2.2.4.2 Secondary outcomes

The secondary outcomes of the study are mainly to explore the prostate cancer-specific mortality of patients diagnosed with COVID-19.

### 2.3 Information sources and search strategy

We aim to search all relevant literature from the date of establishment of each electronic database to September 2020, and the Wanfang database, the China Science Journal Citation Report (VIP database), the China National Knowledge Infrastructure (CNKI), Web of Science, EMBASE, PubMed, and the Cochrane Library will be included. The search strategy of MeSH words and free words is as follows: (“novel coronavirus pneumonia” OR “novel coronavirus-infected pneumonia” OR “NCIP” OR “2019 novel coronavirus” OR “2019-nCoV” OR “corona virus disease 2019” OR “COVID-19”) AND (“prostate tumor” OR “prostate cancer” OR “malignancy”) AND “risk factor.”

### 2.4 Screening

We will arrange for two reviewers to individually filter the duplicate and irrelevant articles in the search results and, then, to delete the content of these duplicate and irrelevant articles for the next analysis. Filtering the remaining results will further obtain the full text and original data of the article, and the following types of articles and data will be eliminated: comments, case reports, letters, and editorials. We will report on the consensus for the collected evidence and consult a third examiner when necessary.

### 2.5 Data extraction

The studies retrieved during the searches will be screened for relevance, and only those studies meeting the standards will be included. After the studies are filtered, we will extract the data from most studies. The following data will be extracted from the selected articles: authors, year of publication, study location, type of study, sample size and age, patient characteristics, duration of intervention, therapeutic scheme, follow-up time, main variables, and main results. Two reviewers will be responsible for extracting and managing the data, which will be inserted into an EXCEL spreadsheet, and doubts will be clarified with the help of a third researcher.

### 2.6 Methodological quality assessment

We will independently assess any bias in the included studies according to the criteria from the Cochrane Handbook, version 5.3.0, which includes random sequence generation, allocation concealment, blinding of participants and personnel, blinding of outcome assessment, incomplete outcome data, selective reporting, and other sources of bias. The quality of each trial will be categorized into a low, unclear, or high risk of bias. We will resolve any differences in opinion through discussion or consultation with the third author. The overall quality of this systematic review and meta-analysis will be summarized and evaluated with GRADEpro software (http://www.gradepro.org).

### 2.7 Data analysis

We will use Review Manager 5.3 software for meta-analysis. Dichotomous data will be presented as odds ratio (OR), and continuous data calculated using weighted mean deviations (WMDs) or standardized mean deviations (SMDS), and both will have corresponding 95% confidence intervals (CIS). Statistical heterogeneity will be assessed by the *P*-value and *I*^2^ statistic. For values of *P* > .05, and *I*^2^ ≤ 50%, a fixed-effect model will be selected for use; however, if *P* ≤ .05, and *I*^2^ > 50%, a random-effect model will be selected instead.

## 3 Discussion

COVID-19 has become a global public health emergency. Coronavirus can bring about diversified systemic infections in multiple animals and mainly leads to respiratory tract infections in humans, and mortality from coronavirus is very high in patients with severe acute respiratory syndrome (SARS).^[11,12]^ SARS and Middle East respiratory syndrome (MERS) are also caused by coronaviruses.^[13,14,15]^ Respiratory failure is the main cause of death related to SARS and MERS; however, multiple organ dysfunction syndrome is the main cause of death from COVID-19.^[16]^

At present, there are no drugs approved in any country for the prevention or treatment of COVID-19. Studies have shown that remdesivir, chloroquine, and hydroxychloroquine can inhibit the in vitro growth of SARS-CoV-2, and they are expected to work in vivo.^[17,18,19]^ Chloroquine and hydroxychloroquine are antimalarial agents and have been prescribed for the treatment of autoimmune diseases (e.g., rheumatoid arthritis and lupus) for almost 70 years.^[20]^ Based on the results of in vitro studies, chloroquine and hydroxychloroquine have been used in some clinical trials to prevent and treat COVID-19. However, the effects of chloroquine and hydroxychloroquine are still not completely understood.^[21]^ Recent studies show that hydroxychloroquine did not show a significant difference in preventing the incidence rate of COVID-19 compared with a placebo.^[22]^ Moreover, the use of hydroxychloroquine and other drugs may lead to prolonged QT interval and drug-related sudden cardiac death.^[23]^

Because the tumor itself and some anti-tumor therapies may lead to systemic immunosuppression in tumor patients, cancer patients more prone to infection than nontumor patients.^[24–27]^ More importantly, compared with cancer patients, tumor patients have a higher risk of SARS-CoV-2 infection and a worse prognosis. Among them, male patients with cancer are more likely to be infected with SARS-CoV-2 and have a worse prognosis.^[28]^ In a study by Montopoli et al, in which only male patients were analyzed, the proportion of cancer patients was 9.5% (430/4532), while prostate cancer patients accounted for 2.6% (118/4532).^[10]^

So far, no vaccines or drugs have been approved for the remedy of COVID-19, and neutralizing antibodies with strong specificity are considered to be potential “special effect drugs” for COVID-19 therapy. Humanized neutralizing antibodies provide new hope for fighting COVID-19, as they prevent the coronavirus from binding with human cells.^[29–31]^ Although many experts around the world are rapidly developing a coronavirus vaccine, it will take at least 12 to 18 months from vaccine development to human application. Therefore, the prevention of COVID-19 may be more important than the treatment. The epidemic is raging around the world, and it is clear that prostate cancer is a significant hazard factor for men infected with SARS-CoV-2. This knowledge can better maintain male health and guide clinicians to prevent COVID-19 in prostate cancer patients.

## Author contributions

**Data curation:** Wenjie Li, Zhao Liu.

**Formal analysis:** Mingxin Wu, Xiaodi Liu.

**Funding acquisition:** Xiaojiang Li, Yingjie Jia.

**Methodology:** Shanqi Guo, Xinyao Jin.

**Project administration:** Wenjie Li.

**Resources:** Xiaojiang Li, Yingjie Jia.

**Software:** Xiaodi Liu, Xinyao Jin.

**Writing – original draft:** Mingxin Wu, Ruiyu Mou.

**Writing – review & editing:** Ruiyu Mou, Shanqi Guo, Wenjie Li.
